# Breaking the proteasome balance: α-synuclein and the ubiquitin-proteasome system

**DOI:** 10.3389/fcell.2026.1906331

**Published:** 2026-09-10

**Authors:** Tariq T. Ali, Blagovesta Popova, Gerhard H. Braus

**Affiliations:** 1 Department of Molecular Microbiology and Genetics, Institute of Microbiology and Genetics, University of Göttingen, Göttingen, Germany; 2 Department of Genetics of Eukaryotic Microorganisms, Institute of Microbiology and Genetics, University of Göttingen, Göttingen, Germany

**Keywords:** 20S proteasome, 26S proteasome, alpha-Synuclein, autophagy, Parkinson disease, posttranslational modifications, protein homeostasis, UPS

## Abstract

Aging is the major risk factor for synucleinopathies, including Parkinson’s disease, dementia with Lewy bodies, and multiple system atrophy. However, the molecular mechanisms linking aging to. α-Synuclein cytotoxicity remain incompletely understood. The progressive decline of proteostasis is central among these mechanisms, as the ubiquitin-proteasome system (UPS) and the autophagy-lysosome pathway fail to maintain the turnover of aggregation-prone proteins. In this review, we focus on the bidirectional crosstalk between α-Synuclein and the proteasome as a key driver of proteostasis collapse in synucleinopathies. Proteasome activity declines during aging, and proteasomal dysfunction is closely associated with disease progression. We first discuss how. α-Synuclein structure and posttranslational modifications determine whether the protein is targeted for degradation by the UPS or autophagy or instead acts as a proteolytic inhibitor. Next, the mechanisms by which pathogenic α-synuclein species interact with and impair 20S/26S proteasomes, affecting proteolytic activity, subunit composition, and complex assembly, are discussed. These interactions establish a self-amplifying cycle of proteasome inhibition and α-Synuclein accumulation. As a consequence, the cellular capacity to clear misfolded proteins progressively declines, promoting toxic α-Synuclein aggregation and neurodegeneration. We further discuss the importance of autophagy on α-Synuclein turnover and how this is impaired in synucleinopathies. Finally, we highlight emerging therapeutic strategies aimed at restoring proteasome function and proteostasis. A deeper understanding of how α-Synuclein-proteasome interactions change during ageing may reveal new molecular targets and support the development of disease-modifying therapies for synucleinopathies.

## 1 Introduction

Maintaining proteostasis is a key prerequisite for cellular function and survival. Proteostasis comprises the regulated synthesis, folding, localization and degradation of proteins. Aging results in and is reinforced by the breakdown of key cellular processes, referred to as hallmarks of aging (López-Otín et al., 2023). Loss of proteostasis, alongside disrupted autophagy and mitochondrial dysfunction are the key metabolic processes, which are progressively impaired during aging. The outlined breakdown is compounded by the dysregulation of other cellular and inter-cellular pathways, such as senescence, telomere attrition and chronic inflammation. The resulting impairment of cellular homeostasis leads to the accumulation of reactive oxygen species (ROS), misfolded and aggregated proteins, and alterations in gene expression, among other cellular aberrations (Hipp et al., 2019; Płóciniczak et al., 2025; An et al., 2025).

Age-related diseases, including Parkinson´s disease (PD), are closely linked to the decline in cellular homeostasis. In PD, the most prominent connection between aging and disease is the accumulation of aggregated α-Synuclein (αSyn). Under physiological conditions, this small, natively unfolded protein contributes to vesicle trafficking and fusion at the presynaptic terminal, thereby supporting neurotransmitter release (Sharma and Burré, 2023). αSyn has also been linked to axonal vesicle transport, mitochondrial homeostasis, transcription modulation and epigenetic modulation through interaction with histones (Barba et al., 2022; Surguchov, 2023). Its aberrant accumulation therefore has the potential to disrupt several cellular processes, which simultaneously reflect established hallmarks of aging. Rather than affecting a single pathway, pathological αSyn influences multiple interconnected cellular processes that collectively determine proteome integrity and neuronal viability. Aggregated αSyn impairs both autophagy and the ubiquitin-proteasome system (UPS), two major proteolytic pathways responsible for clearing misfolded, damaged, and accumulated proteins (Fellner et al., 2021; Sahoo et al., 2022). Inhibition of protein degradation systems exacerbates the aggregation of αSyn into polymorphous aggregates and amyloid fibrils, a hallmark of PD and other synucleinopathies, such as Dementia with Lewy Bodies (DLB) and Multiple System Atrophy (MSA) (Srinivasan et al., 2021). Not only Synucleinopathies represent a broad field of disorders with shared pathomechanisms. PD itself includes a variety of forms, which further complicates research for gaining therapeutic approaches (Outeiro et al., 2023). This reciprocal relationship establishes a self-propelling cycle in which impaired proteostasis promotes αSyn accumulation. Increased aSyn abundance then further compromises cellular protein degradation pathways. Although substantial experimental evidence supports this concept, the relative contribution of individual mechanisms to disease progression in humans remains incompletely understood.

The aggregation of αSyn into amyloid fibrils is a long, multi-step process, initiated by the formation of amorphous oligomers (Mehra et al., 2019). These cytotoxic assemblies can act as seeding structures for the formation of highly ordered amyloid fibrils, which are deposited into Lewy Bodies (LB), the most prominent histological marker for synucleinopathies (Estaun-Panzano et al., 2023). Interestingly, the structure of amyloid fibrils differs between the different synucleinopathies, highlighting the diversity within this group of diseases, which is a key feature relevant to the topic of this review (Yang et al., 2022). Increasing structural evidence further indicates that distinct αSyn conformers differ not only in their architecture but also in their biochemical properties and cellular toxicity. These αSyn interactions with protein quality control systems are outlined throughout this review. These conformational differences are likely to contribute to the heterogeneous effects of αSyn reported across experimental studies and may underlie disease-specific pathogenic mechanisms.

Aggregated αSyn species are not only cytotoxic but also interfere with protein degradation pathways (Lindersson et al., 2004; Fellner et al., 2021; Sahoo et al., 2022). Under physiological conditions, αSyn stabilizes the soluble N-ethylmaleimide-sensitive-factor attachment receptor (SNARE) protein SNAP29, which facilitates vesicle fusion. Elevated αSyn levels reduce SNAP29 abundance, thereby impairing the autophagosome-lysosome fusion and autophagic flux (Tang et al., 2021). The autophagic deficit is compounded by an impaired omegasome formation, resulting in fewer autophagosomes (Winslow et al., 2010). In parallel, pathological αSyn impairs the UPS through several mechanisms, including defective 26S assembly (Galka et al., 2024), altered proteasomal subunit abundance (Popova et al., 2021), reduced catalytic activity (Thibaudeau et al., 2018), and direct interactions with proteasomal subunits (Ghee et al., 2000; Snyder et al., 2003; Alvarez-Castelao et al., 2014; Popova et al., 2021). In addition, mutations in genes linked to UPS regulation, including *PARK2* and *PARK15* in familial cases of PD further support the relevance of UPS dysfunction in the pathogenesis of Parkinson´s disease. However, the precise molecular mechanisms linking αSyn pathology to autophagic and UPS dysfunction remain an active area of investigation. Experimental findings differ considerably depending on the αSyn species examined, the proteasome complexes investigated, and the experimental model employed. This supports that the relationship between αSyn and the protein degradation machineries can vary in a context-dependent manner.

This review summarizes current knowledge on the interplay between αSyn and the UPS, with particular emphasis on αSyn as both a proteasomal substrate and a proteasome inhibitor.

## 2 The Parkinson’s-associated protein α-synuclein

αSyn is a 140-amino-acid protein that exists in a natively unfolded state in the cytoplasm under physiological conditions. The key pathological hallmark of PD is the accumulation of aggregated αSyn species, including amyloid fibrils, that are deposited into LB or Lewy neurites (LN). It belongs to the synuclein family, which also includes β-synuclein and γ-synuclein. All three proteins are natively unstructured in the cytoplasm and are predominantly found in neuronal tissues and are primarily localized to the presynaptic terminal (George, 2001).

### 2.1 Sequence and structure of α-synuclein

**FIGURE 1:**
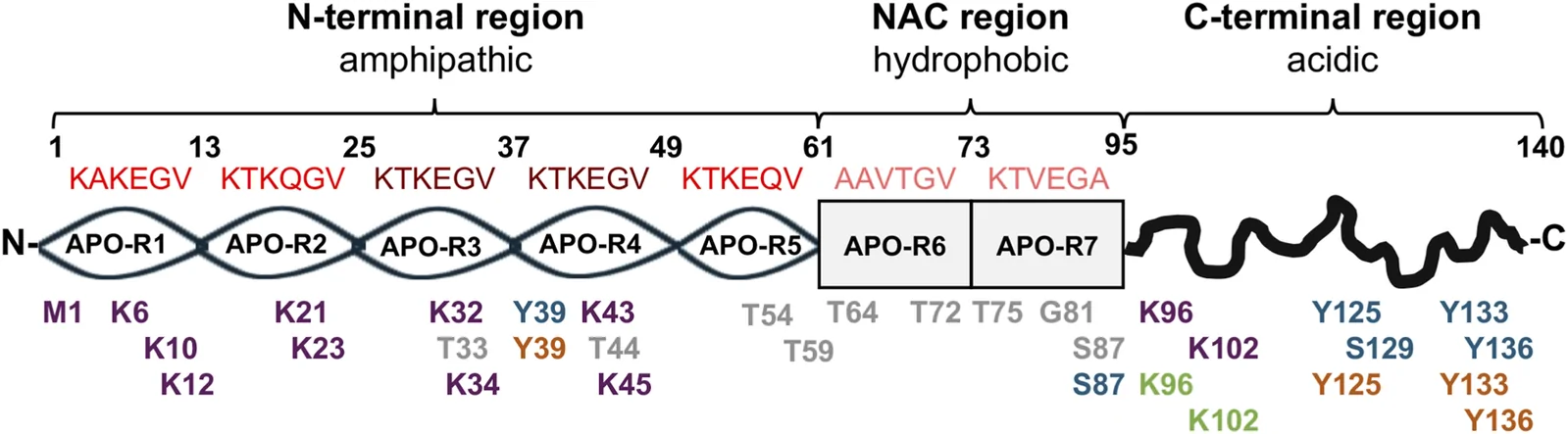
α-Synuclein protein domains including posttranslational modification sites and apolipoprotein-lipid-binding motifs (APO) repeats. The amphipathic N-terminal region contain of five apolipoprotein repeats (APO-R1-5). Two additional less conserved APO motifs constitute the hydrophobic non-amyloid-β-component (NAC) region, associated with αSyn aggregation. APO motifs are coloured red, with dark red shades indicating perfect repeats of the KTKEGV-motif. Lighter shades of red indicate lower degrees of conservation. Following the NAC region is the acidic unfolded C-terminal region of αSyn. Posttranslational modifications are colour-coded, highlighting ubiquitination (violet), SUMOylation (green), phosphorylation (blue), O-GlcNAcylation (grey) and nitration (orange).

αSyn adopts an α-helical secondary structure upon interacting with membranes under physiological conditions, contributing to vesicle fusion. In pathological states, αSyn undergoes conformational misfolding, forming β-sheet-rich structures that predispose it to aggregation. The structure of αSyn, including posttranslational modifications (PTMs) is shown in Figure 1.

The ability of αSyn to interact with membranes is based on the seven imperfect apolipoprotein-lipid-binding motifs (APO), which fold into amphipathic helices upon interaction with a membrane (Brontesi et al., 2023). Five of the seven APO-repeats are located in the amphipathic membrane-binding N-terminus of the protein (Stefanis, 2012). The non-amyloid-β-component (NAC) domain and the acidic C-terminus are not involved in the interaction with membranes. Many disease-associated mutations result in N-terminal amino acid exchanges (Manzanza et al., 2021). A large abundance of valine and alanine amino acid residues result in a highly hydrophobic NAC-region of αSyn. This hydrophobicity is a key driver in misfolding of the protein into pathological β-sheet structure. The APO-repeats within this domain are less conserved in comparison to the APO-motifs located in the N-terminus (Brontesi et al., 2023). Finally, the C-terminus remains unfolded both in physiological membrane-bound α-helices, as well as pathological β-sheets. Although it does not form the amyloid core, the C-terminus is rich in PTMs that influence aggregation propensity and degradation. Overall, the structural organization of αSyn provides a mechanistic framework for understanding how disease-associated DNA mutations and posttranslational amino acid modifications differentially influence protein behavior. Rather than acting independently, the three structural domains exhibit strong functional interdependence. Modifications within one domain can alter the conformational dynamics and accessibility of distant regions. αSyn is a highly dynamic protein, whose interactions with membranes, molecular chaperones, and protein degradation machineries are continuously shaped by its conformational state. This structural plasticity likely contributes to the considerable variability observed between experimental studies investigating αSyn aggregation and degradation.

### 2.2 Posttranslational α-synuclein modifications

PTMs are unevenly distributed throughout αSyn. Ubiquitination sites are concentrated mainly in the N-terminal region of the protein, whereas the NAC region contains comparatively few PTMs, with the exception of S87 phosphorylation/nitration and several O-GlcNAcylation sites (Manzanza et al., 2021). The acidic C-terminus is highly modified with multiple sites for phosphorylation, nitration, as well as residues capable to accepting small ubiquitin-like modifiers (SUMO) (Figure 1). As PTMs are highly important for the aggregation of αSyn and the subsequent loss of proteolytic potential, we seek to outline the most important PTMs of αSyn and how they influence aggregation as well as degradation of the protein.

Phosphorylation is one of the best-studied PTMs of αSyn in the context of PD. Four phosphorylated tyrosine residues (pY39/125/133/136) (Chen et al., 2009; Dikiy et al., 2016; Sano et al., 2021), and two phosphorylated serine residues (pS87/129) (Anderson et al., 2006; Paleologou et al., 2010) have been identified. Especially pS129 has received particular attention, as this residue is abundantly phosphorylated in brains of patients with PD, multiple system atrophy and other synucleinopathies but detected only at low levels in healthy controls (Fujiwara et al., 2002; Oueslati, 2016).

Whereas the level in cells with αSyn-pathology of pS87 or pS129 are significantly increased in cells with αSyn-pathology, phosphorylated αSyn is often less prone to aggregation (Paleologou et al., 2010; Tenreiro et al., 2014; Ghanem et al., 2022). This contradiction suggests that at least some phosphorylation events may occur after initial aggregation or may limit further fibril growth rather than prevent the first nucleation step. Residue-specific phosphorylation modulates αSyn aggregation through different mechanisms. Phosphorylation at S129 occurs after aggregation and appears to reduce the likelihood of further αSyn accumulation (Ghanem et al., 2022). Phosphorylation at S87 might prevent fibrilization, although it remains unclear whether this effect occurs before or after the initial aggregation process (Paleologou et al., 2010). The anti-aggregation effect of pS87 may be attributed to its location in the hydrophobic NAC region of the protein. By introducing a negative charge to this region, phosphorylation disrupts the hydrophobic stretch necessary for fibril formation, an effect that can be mimicked by the phosphor-mimicking mutant S87 E (Oueslati et al., 2012). A similar aggregation-reducing effect is observed for pS129. However, the mechanism might differ: the acidic αSyn C-terminus where S129 resides, is not directly involved in amyloidogenesis and remains unfolded during fibrilization. pS129 might exert its effects in a manner distinct from pS87. Notably, the C-terminus of αSyn plays a role in various interactions, including protein-protein, protein-ligand, and protein-metal binding, suggesting potential physiological functions for C-terminal phosphorylation sites, including both serine and tyrosine residues (Burré et al., 2010; Shvadchak et al., 2011; Lautenschläger et al., 2018; Sharma and Burré, 2023).

Physiological tyrosine phosphorylation is more common for αSyn and strongly linked to anti-aggregation functions. Y39 phosphorylation reduces αSyn aggregation propensity and promotes a partial-helix conformation, which may differentially influence aggregation dynamics *in vivo* (Dikiy et al., 2016). Similarly, pY125 exhibits reduced aggregation (Chen et al., 2009; Hejjaoui et al., 2012). Y136 phosphorylation also prevents aggregation, whereas the pY133 effect on aggregation is poorly understood (Sano et al., 2021). Tyrosine residues can also be nitrated, which will be expanded more upon later. The phosphorylation of these serine and tyrosine residues is mediated by a variety of kinases, as detailed in Table 1. Phosphorylation of αSyn modulates its aggregation propensity but also significantly influences its degradation. pS129 is preferentially proteasome degraded compared to non-phosphorylated αSyn and can enhance autophagic clearance (Sandoval-Pistorius et al., 2023; Tenreiro et al., 2014). Tyrosine phosphorylation of αSyn at Y136 promotes lysosomal degradation and association with heat shock protein 70 (Choi et al., 2012), whereas phosphorylation at Y39, and, to a lesser extent, Y125 by the c-Abl tyrosine kinase impairs both lysosomal and proteasomal degradation of αSyn (Mahul-Mellier et al., 2014). Thus, phosphorylation influences not only aggregation but also the route and efficiency of αSyn turnover. An overview of the effects of different posttranslational modifications on αSyn has been given in Table 2. Collectively, these findings illustrate that phosphorylation cannot simply be classified as either protective or pathogenic. Instead, its biological consequences appear to depend on the modified residue, the temporal sequence of phosphorylation relative to aggregation, and the cellular context in which it occurs. This context dependency probably explains why phosphorylation has been reported to either suppress or promote αSyn pathology in different experimental systems. Furthermore, many mechanistic insights originate from biochemical studies or overexpression models, whereas considerably less is known about the precise temporal dynamics of phosphorylation during human disease progression. Future studies integrating structural biology with longitudinal analyses in physiological models will therefore be essential to define the causal contribution of individual phosphorylation events to synucleinopathies.

**TABLE 1:** Known kinases of αSyn and their target residues.

Residue	Kinase	Source
S87	Casein Kinase I (CKI)	(Okochi et al., 2000)
S129	Casein Kinase I/II (CKI/II)	(Okochi et al., 2000)
	G-Protein-Coupled Receptor Kinase 1/2/5/6 (GRK1/2/5/6)	(Pronin et al., 2000)
	Leucine Rich Repeat Kinase 2 (LRRK2)	(Qing et al., 2009)
	Polo-Like Kinase 2 (PLK2)	(Inglis et al., 2009)
	Protein Kinase C-Related Kinase (PRK)	(Reimer et al., 2018)
	MAP Kinase-Interacting Kinase 2a (Mnk2a)	(Zhang et al., 2015)
Y39	c-Ableson Tyrosine Kinase (c-Abl)	(Mahul-Mellier et al., 2014)
Y125	Spleen Associated Tyrosine Kinase (Syk)	(Negro et al., 2002)
	c-Fgr	(Negro et al., 2002)
	Lyn	(Negro et al., 2002)
	Fyn	(Nakamura et al., 2001)
	c-Ableson Tyrosine Kinase (c-Abl)	(Mahul-Mellier et al., 2014)
Y133	Spleen Associated Tyrosine Kinase (Syk)	(Negro et al., 2002)
Y136	Spleen Associated Tyrosine Kinase (Syk)	(Negro et al., 2002)

**TABLE 2:** Impact of different posttranslational modifications on the degradation of αSyn. Residues titled “overall” indicate the effect of the modification has been determined generally, without verification of specific residues.

Ptm	Residue	Effect	System	Citation
Phosphorylation	Y39	Decrease	Autophagy + UPS	(Mahul-Mellier et al., 2014)
	Y125	Decrease	Autophagy + UPS	(Mahul-Mellier et al., 2014)
	Y136	Increase	Autophagy	(Choi et al., 2012)
	S129	Increase	Autophagy	(Tenreiro et al., 2014)
Nitration	Y39	Decrease	Autophagy + UPS	(Hodara et al., 2004)
	Y125	Decrease	Autophagy + UPS	(Stykel and Ryan, 2022)
	Y133	Decrease	Autophagy + UPS	(Stykel and Ryan, 2022)
	Y136	Decrease	Autophagy + UPS	(Stykel and Ryan, 2022)
Glycosylation	overall	Increase	Autophagy	(Miao et al., 2025)
	T72	Decrease	UPS	(Levine et al., 2019)
	S87	Decrease	UPS	(Levine et al., 2019)
Mono-Ubiquitination	K10	Increase	UPS	(Rott et al., 2011)
	K12	Increase	UPS	(Rott et al., 2011)
	K21	Increase	UPS	(Rott et al., 2011)
	K23	Increase	UPS	(Rott et al., 2011)
	K34	Increase	UPS	(Rott et al., 2011)
	K43	Increase	UPS	(Rott et al., 2011)
	K96	Increase	UPS	(Rott et al., 2011)
Poly-Ubiquitination	M1	Increase	Autophagy	(Furthmann et al., 2023)
	K12	Increase	Autophagy	(Tofaris et al., 2011)
	K21	Increase	Autophagy	(Tofaris et al., 2011)
	K45	Increase	Autophagy + UPS	(Ho and Wing, 2024)
	K58	Increase	Autophagy + UPS	(Ho and Wing, 2024)
	K60	Increase	Autophagy + UPS	(Ho and Wing, 2024)
	K96	Increase	Autophagy	(Tofaris et al., 2011)
SUMOylation	overall	Decrease	Autophagy + UPS	(Rott et al., 2017)

In addition to phosphorylation, αSyn undergoes tyrosine nitration under conditions of oxidative and nitrosative stress. Reactive nitrogen species (RNS) can nitrate the same tyrosine residues that are targets for phosphorylation (Y39, Y125, Y133, and Y136), resulting in the formation of 3-nitrotyrosine (Bartesaghi and Radi, 2018). In PD, mitochondrial dysfunction, ROS accumulation, and increased nitric oxide synthase activity can promote this modification (Tsang and Chung, 2009; Stykel and Ryan, 2022). Unlike phosphorylation, which requires ATP and specific kinase activity, tyrosine nitration is a radical-mediated reaction.

This modification has significant implications for disease pathogenesis. Nitrated αSyn exhibits increased propensity for fibril formation, and impaired proteasomal degradation, further exacerbating protein accumulation and aggregation (Hodara et al., 2004). C-terminal nitration has also been linked to enhanced clearance of αSyn inclusions through autophagy. Interestingly, either nitration or phosphorylation of Y133 is a pre-requisite for phosphorylation of S129 and thus influences αSyn aggregation and degradation (Kleinknecht et al., 2016). Even more broadly speaking, tyrosine nitration plays a dual role, possibly contributing to pathology in a context-dependent manner by altering both αSyn aggregation and clearance. The extensive crosstalk between these modifications adds another level of complexity and may contribute to the heterogeneous biochemical properties of αSyn species isolated from different experimental models and human synucleinopathies.

The NAC region also contains several O-linked β-N-acetylglucosamine (O-GlcNAcylation) modification sites. Because other forms of glycosylation are not central to this review, O-GlcNAcylation will be referred to as glycosylation. Interestingly, besides pS87, all other PTMs described in the NAC region are O-GlcNAcylation sites (Manzanza et al., 2021). Among the nine described glycosylation sites, eight are threonine residues, in addition to the previously mentioned S87 (Levine et al., 2019). Glycosylation is often associated with facilitating accurate cellular localization of proteins and tissue-specific knockout of glycosylating enzymes can result in neurodegeneration and neuronal death (Wang et al., 2016). Glycosylation of αSyn does not significantly alter its monomeric structure but reduces its aggregation propensity (Zhang et al., 2017) and neuronal toxicity (Levine et al., 2019). This effect appears strongest when threonine residues are modified and likely occurs during early nucleation steps. It is important to distinguish enzymatic glycosylation from non-enzymatic glycosylation (also referred to as glycation), a process, where reducing sugars covalently bind to amino acids with free amine groups. Non-enzymatic glycation of lysine residues in the N-terminus decreases lipid binding, enhances aggregation, and aggravates synuclein-associated phenotypes (Vicente Miranda et al., 2017).

Ubiquitination of proteins by various ubiquitination motifs for various functions are key mechanisms which allow accurate for correct protein localization. This review focuses for αSyn and the relevant ubiquitin signals that direct αSyn towards the proteasome or autophagy. K48-linked polyubiquitin is classically associated with proteasomal degradation, whereas K11, K27, and K29 linkages can also contribute to proteasomal targeting (Grumati and Dikic, 2018; Manzanza et al., 2021; Dongdem et al., 2024). It is also important to note that monoubiquitination and short K48-or K63-linked ubiquitin chains may also function as autophagy signals (Cen et al., 2023). αSyn contains multiple ubiquitination sites, mainly within its membrane-interacting N-terminal region. Additionally, two lysine residues in the C-terminal region have been identified as targets for both ubiquitination or SUMOylation.

A variety of ubiquitinating and de-ubiquitinating enzymes regulate αSyn. The really interesting new gene (RING)-like E3 ubiquitin ligases SIAH-1 (seven in absentia homolog-1) and SIAH-2 monoubiquitinate αSyn at residues K10, K12, K21, K23, K34, K43, and K96 (Lee et al., 2008). Monoubiquitinated αSyn has been detected in LB and LN (Tofaris et al., 2003), suggesting that ubiquitination may enhance aggregation (Lee et al., 2008; Zenko et al., 2023). However, other studies indicate that monoubiquitinated αSyn is efficiently degraded by the proteasome, and deubiquitination enhances aggregation (Rott et al., 2011). This apparent discrepancy may reflect a dual role in which monoubiquitinated monomers are efficiently degraded, while ubiquitination of fibrillar αSyn does not guarantee clearance (Nonaka et al., 2005). These conflicting findings illustrate that ubiquitination should not be viewed as a universal degradation signal. Instead, the biological outcome depends on multiple parameters, including ubiquitin linkage type, chain length, substrate conformation, and the cellular degradation machinery available. Monomeric, oligomeric, and fibrillar αSyn differ substantially in their accessibility to ubiquitination enzymes and proteolytic systems, which likely explains many of the discrepancies reported in the literature. Consequently, ubiquitination appears to function less as a binary degradation signal than as a dynamic regulatory code that determines the intracellular fate of distinct αSyn species.

Other ubiquitin linkages appear to route αSyn toward lysosomal or combined degradation pathways. The E3 ligases NEDD4 *(*neural-precursor-cell-expressed developmentally downregulated 4) and NEDD4-1 poly-ubiquitinate αSyn at residues K12, K21, K45, K58, K60, and K96 through K63-linked polyubiquitin chains, promoting the protein uptake and degradation (Tofaris et al., 2011). Branched K48/K63 ubiquitin chains can also be added to fibrillar αSyn by the Cullin-RING-ligase SCF-FBXL5. This ligase contains S-phase associated protein 1, cullin-1 as well an F-box-domain containing protein (SCF). The SCF itself is associated with the F-box/LRR-repeat protein 5 (FBXL5). (Gerez et al., 2019).

These chains are attached to residues K45, K58, and K60 of αSyn, with the SCF complex facilitating degradation of the ubiquitinated protein via both lysosomal and proteasomal pathways (Ho and Wing, 2024). *In vitro*, K48-linked chains are added preferentially to monomeric αSyn at residues K21, K23, K32, and K34, whereas fibrillar αSyn is ubiquitinated less efficiently and at residues K6, K10, and K12 (Nonaka et al., 2005). Poly-ubiquitinated αSyn, particularly tetra-ubiquitinated αSyn at residue K12, exhibits a higher propensity to form amorphous aggregates *in vitro* but not mature amyloid fibrils (Haj-Yahya et al., 2013). Simultaneously, this ubiquitin linkage enhances proteasomal degradation of the protein (Haj-Yahya et al., 2013). Finally, ubiquitination of the M1 residue has been studied, involving a linear ubiquitin chain linked via the K63 residue of ubiquitin. The Linear Ubiquitin Chain Assembly Complex (LUBAC) recruits the NF-κB-essential modulator (NEMO) protein to ubiquitinate αSyn aggregates at M1, promoting their lysosomal degradation (Furthmann et al., 2023). Taken together, these studies demonstrate that different ubiquitin architectures selectively direct αSyn toward proteasomal degradation, autophagy, or combined degradation pathways. However, the extent to which these mechanisms operate in human neurons under physiological expression levels remains incompletely understood, as much of the current evidence derives from *in vitro* reconstitution assays and cellular overexpression systems.

SUMOylation adds another layer of regulation. αSyn contains several SUMOylated lysine residues, with K96 and K102 being particularly relevant (Krumova et al., 2011). Early studies suggested that SUMOylation reduces fibril formation and cytotoxicity (Krumova et al., 2011). Subsequent work in cellular models demonstrated an interplay between sumoylation and phosphorylation in regulating αSyn turnover. In this context, SUMOylation exerted a protective effect against αSyn toxicity and inclusion formation, while defects in SUMOylation could be partially compensated by phosphorylation (Shahpasandzadeh et al., 2014). More recent studies, however, have implicated SUMOylation as a driver of pathology. In rat brain extracts, SUMOylation of αSyn blocks its monoubiquitination, thereby reducing proteasomal degradation, whereas blocked polyubiquitination by SUMO interferes with autophagy. Additionally, SUMOylated αSyn is more readily exported from cells, potentially contributing to the spreading of pathology. Consistent with this, elevated levels of SUMOylated αSyn have been detected in the brains of PD patients (Rott et al., 2017). The seemingly contradictory effects of SUMOylation likely reflect differences in experimental models, the specific SUMO isoforms involved, and the conformational state of αSyn undergoing modification. Current evidence therefore suggests that SUMOylation is neither exclusively protective nor pathogenic but rather acts as a context-dependent regulator of αSyn homeostasis. Defining how SUMOylation interacts with ubiquitination, phosphorylation, and other PTMs will be essential for understanding how combinations of PTMs collectively determine αSyn stability, aggregation, and degradation.

## 3 The ubiquitin proteasome system

### 3.1 Architecture and assembly of the proteasome

The 26S proteasome is the main ATP-dependent proteolytic complex responsible for selective degradation of ubiquitinated proteins in eukaryotic cells. It consists of a central 20S core particle (CP) capped with one or two 19S regulatory particles (RPs) (Bard, 2018). The CP is composed of four stacked heptameric rings: two outer α-rings that regulate substrate entry and two inner β-rings that contain the catalytic sites. The entrance to the proteolytic chamber is normally occluded by the by the N-termini of several α-subunits and is stabilized by conserved tyrosine-aspartic acid-arginine (YDR) motifs (Groll et al., 1997; Chuah et al., 2023) Proteolysis is mediated by the β1, β2, and β5 subunits, which provide caspase-like, trypsin-like, and chymotrypsin-like activities, respectively (Tomko and Hochstrasser, 2013). Although the structural organization of the proteasome has been extensively characterized, it is increasingly recognized that the proteasome should not be regarded as a static degradation machine. Instead, proteasome composition, assembly, and regulator association are highly dynamic processes that adapt to cellular stresses, various metabolic states, and substrate availabilities. This dynamic remodeling has emerged as an important concept in neurodegeneration, where alterations in proteasome composition may influence the capacity to degrade aggregation-prone proteins such as αSyn.

**FIGURE 2:**
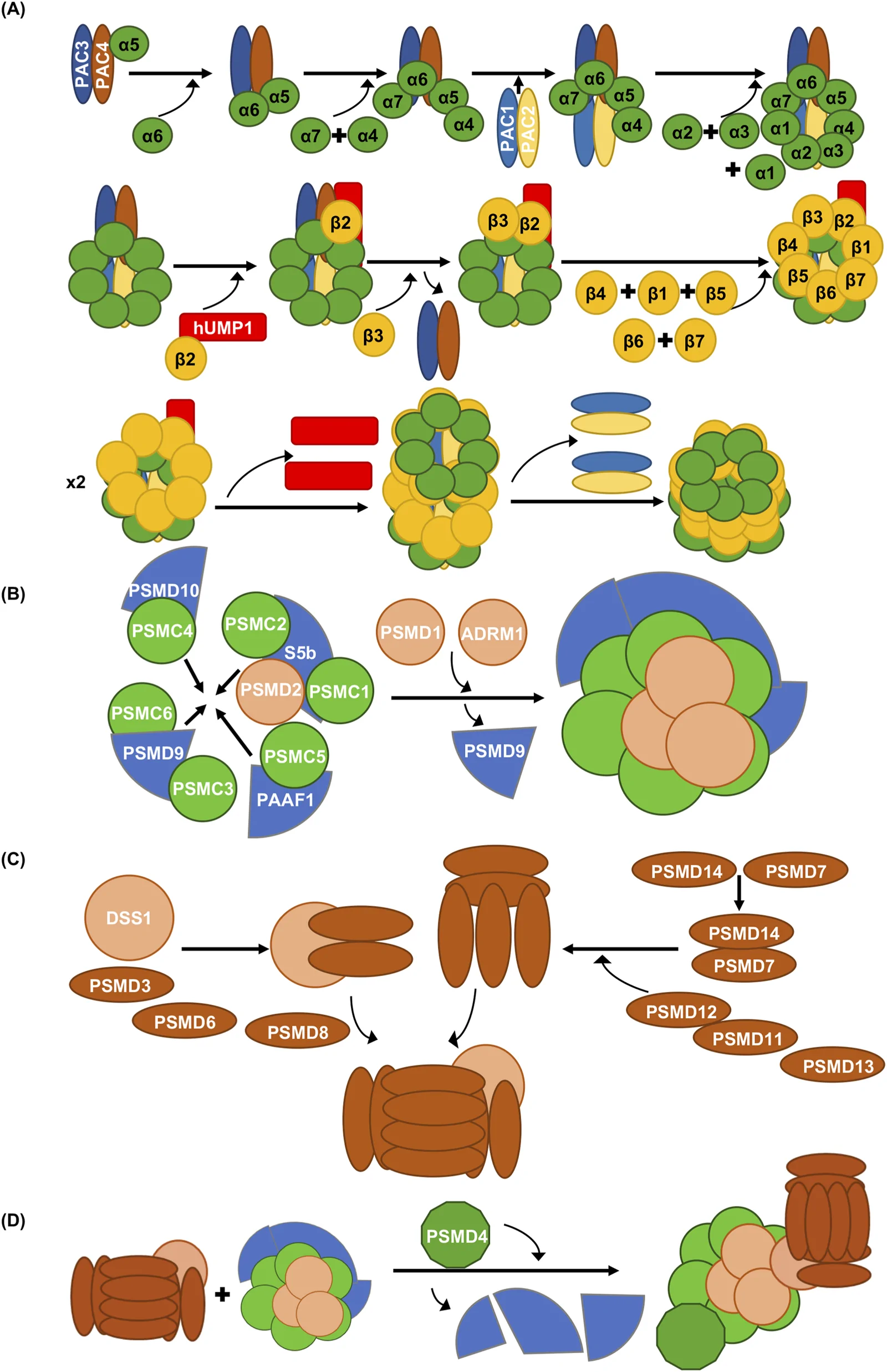
Assembly of the proteasome core particle and regulatory particle. **(A)** Assembly of the 20S proteasome core particle. Assembly begins with the recruitment of α5 to the PAC3/PAC4 heterodimeric chaperone complex. This is followed by recruitment of α6 and the subsequent addition of α7 and α4. Following the incorporation of the second heterodimer, PAC1/PAC2, the final subunits of the α-ring are incorporated. Addition of the chaperone hUMP1, loaded with the β2 subunit initiates the formation of the β-ring. This requires the dissociation of PAC3/PAC4 before the remaining β-subunits can be added to the nascent 13S half-proteasome. Two half-proteasomes together form the final 20S mature proteasome, while the remaining chaperones are removed. **(B)** Assembly of the 19S regulatory particle base. Chaperones (blue) bind individual or multiple base subunits prior to assembly of the base. Subunits shown in green are AAA + -ATPases, while the orange subunits are non-ATPase subunits. In particular, ADRM1 and PSMD2, are involved in the binding of ubiquitin and ubiquitin-like proteins to the proteasome. After the assembly of the base, two chaperones PAAF1 and S5b remain bound to the base until the regulatory particle is formed. **(C)** Assembly of the 19S regulatory particle lid complex. The assembly of the lid particle does not require dedicated chaperones. Instead, the lid pre-assembles in two separate steps prior to combining. All subunits are non-ATPases, with PSMD14 serving as the intrinsic deubiquitinating enzyme of the proteasome. **(D)** Assembly of the 19S regulatory particle. Base and lid complexes associate to form the mature regulatory particle. During this process, the remaining assembly chaperones are released and the ubiquitin-receptor subunit PSMD4 is incorporated, completing formation of the 19S regulatory particle.

Assembly of the 20S core particle (CP) is a multistep process coordinated by several dedicated chaperones. Formation of the α-ring begins with the recruitment of the α5-and α6 subunits by the PAC3/4 chaperone complex (Matias et al., 2025) (Figure 2A). CP assembly of the α-ring assembly is completed by sequential incorporation of all seven α-subunits as well as the PAC1/2 heterodimeric chaperone. (Murata et al., 2009; Takagi et al., 2014). Subsequently, β-subunits are added to the α-ring in a defined order, starting with hUMP1 associated β2, resulting in formation of the 13S half-proteasome (Hirano et al., 2008). Dimerization of two half-proteasomes generates the immature pre-holoproteasome, and removal of assembly chaperones, yields the mature 20S proteasome (Kunjappu and Hochstrasser, 2014). Beyond ensuring correct proteasome biogenesis, regulated assembly provides an additional level of proteostasis control. Increasing evidence suggests that cells actively remodel proteasome populations during stress by altering the abundance of specific regulatory particles and assembly intermediates. Consequently, impaired proteasome function in neurodegeneration does not solely reflect catalytic inhibition but includes also consequences of defective assembly or altered equilibrium between distinct proteasome species.

Degradation of folded proteins requires association of the 20S CP with other complexes or proteins, most commonly the 19S RP. The RP consists of the base and lid subcomplexes. The base contains six AAA + ATPases (PSMC1–6), which drive substrate unfolding and translocation into the CP, as well as additional subunits involved in structural scaffold (PSMD1) and ubiquitin recognition (PSMD2 and ADRM1) (Bard, 2018). Several base subunits contain a conserved C-terminal HbYX-motif (hydrophobic-tyrosine-any amino acid) that promotes opening of the 20S CP gate (Smith et al., 2007). The lid complex contains the important deubiquitinating subunit PSMD14 and mainly consists of structural subunits (DSS1, PSMD3, PSMD6, PSMD7, PSMD8, PSMD11, PSMD12, PSMD13) (Tomko and Hochstrasser, 2013; Bard, 2018). Assembly of the 19S RP requires multiple RP assembly chaperones (RACs) that guide formation of four different intermediate base assemblies. The four precursors assemble as PSMD1 and ADRM1 are incorporated, resulting in the final base complex (Funakoshi et al., 2009; Tomko and Hochstrasser, 2013) (Figure 2B). In contrast, the lid assembles from two independently formed precursor complexes that subsequently combine into the mature structure, not requiring chaperones for their assembly (Tomko et al., 2015; Budenholzer et al., 2017) (Figure 2C). Final RP assembly involves association of lid and base complexes together with removal of assembly chaperones (Figure 2D).

Formation of the functional 26S proteasome depends on interactions between the α-ring of the 20S CP and the 19S RP base. These interactions are mediated by conserved C-terminal HbYX-motifs present in several PSMC subunits and other proteasome activators such as PAN (proteasome activating nucleotidase), PA200 (PSME4), and 11S activators (Chuah et al., 2023). PA200 and Blm10, which is the functionally conserved yeast homolog, binds the α-ring in a similar manner and stabilizes the open-gate conformation to facilitate substrate entry (Guan et al., 2020). Proteasome activators are increasingly recognized as functional determinants of substrate specificity rather than passive gate-opening factors. Different regulatory particles confer distinct biochemical properties on the 20S core particle, thereby expanding the spectrum of proteins that can be degraded. This concept is particularly relevant for intrinsically disordered proteins such as αSyn, whose degradation may depend on the composition of the associated regulatory complex rather than solely on proteasome abundance or catalytic activity.

### 3.2 Ubiquitination and degradation of proteins

Proteins targeted for degradation by the UPS are marked by specific ubiquitination patterns recognized by the 26S proteasome. Linking multiple ubiquitin proteins (Ub) to the ε-amino group of proteinogenic lysine residues directs proteins to different degradation machineries. Polyubiquitination of proteins with a K48-linked motif is the most common signal for proteasomal protein degradation. Ubiquitination is mediated by the sequential action of ubiquitin activating (E1), conjugating (E2), and ligating (E3) enzymes (Wang et al., 2017). Ubiquitinating enzymes are organized in a hierarchal manner, with few E1-like enzymes and an increasing number of E2 and E3 enzymes. The larger number of E2 and particularly E3 enzymes provides substrate specificity within the UPS (George et al., 2018). Importantly, mutations in the genes of E3 ligases *PARK2* (Parkin) and *PARK15* are associated with familial Parkinson’s disease (PD), linking UPS dysfunction to PD pathogenesis (George et al., 2018). In addition, several UPS-related proteins have been identified in Lewy bodies and Lewy neurites, further supporting a role of impaired proteasomal degradation in αSyn pathology (Xia et al., 2008). However, these observations should be interpreted with caution. The presence of UPS components within Lewy bodies demonstrates an association between proteostasis impairment and αSyn pathology but does not establish whether UPS dysfunction represents a primary pathogenic event or develops secondarily as protein aggregates accumulate. Dissecting cause from consequence remains a central challenge in the field. The interplay between αSyn and the UPS will be discussed later in this review.

Ubiquitination begins with ATP-dependent activation of ubiquitin by E1 enzymes, followed by transfer to E2 conjugating enzymes and finally substrate ubiquitination by E3 ligases. E1 activating enzymes form a high energy thioester bond with Ub-AMP, while a second non-covalently bound Ub-AMP molecule accelerates the transfer of the Ub to an E2-conjugating enzyme (Schulman and Wade Harper, 2009). The conjugating enzyme transfers ubiquitin to the target protein via aminolysis (David et al., 2010). However, the activity of the enzyme is purposefully low, unless interacting with an E3-ligase, to ensure only target proteins are ubiquitinated. E3 ligases are divided into three major families, including RING, HECT (homologous to the E6-AP Carboxyl Terminus*)*, and RBR (RING-BetweenRING-RING) ligases, which differ in their mechanisms of ubiquitin transfer (Yuan et al., 2017; Ebadi et al., 2025). Transfer of ubiquitin from E2-enzymes onto the target protein can be achieved by either forming an E3-Ub intermediate (the case in HECT and RBR ligases), or without the need of forming such an intermediate (RING ligases). These three families have recently be expanded by RZ-finger ligases, which function similar to HECT and RBR ligases and RING-Cys-Relay (RCR) ligases, which target tyrosine instead of lysine residues (Ebadi et al., 2025). Notably, Parkin, encoded by *PARK2*, belongs to the RBR family, highlighting the importance of UPS regulation in PD (Koszela et al., 2025).

Efficient proteasomal degradation additionally requires an accessible unstructured region within the substrate protein that can engage the AAA + ATPase motors of the 19S regulatory particle (Prakash et al., 2004). Ubiquitination itself can locally destabilize protein structure and facilitate unfolding, while additional unfoldases such as p97 may assist this process (Beskow et al., 2009; Carroll et al., 2021). Proteins can be degraded in either direction and the process can be stopped by specific signal sequence within the protein (Arkinson et al., 2024). Ubiquitinated substrates are recognized by several receptors of the 19S regulatory particle, including PSMD2, PSMD4, and ADRM1, and subsequently deubiquitinated by PSMD14 or associated deubiquitinases such as USP14 and UCH37 (Tomko and Hochstrasser, 2013; Bard, 2018). Engagement of unfolded substrate regions by the AAA + ATPases commits the substrate to degradation and drives unfolding and translocation into the catalytic chamber of the 20S core particle (Bard et al., 2019). Importantly, successful degradation depends not only on ubiquitin recognition but also on the intrinsic biophysical properties of the substrate. Stable protein domains, aggregation, or reduced accessibility of unstructured initiation regions can markedly decrease degradation efficiency. Consequently, alterations in αSyn conformation during oligomerization and fibrillization, which could result from posttranslational modifications *in vivo*, or differing preparation methods and buffer *in vitro* are expected to influence proteasomal processing independently of ubiquitination status.

Within the 20S core particle, proteolysis is mediated by the three catalytic β-subunits, each possessing either a chymotrypsin-like (β5), trypsin-like (β2) or caspase-like (β1) activity (Thibaudeau and Smith, 2019). This results in the release of short amino acid chains, with the exit of those chains occurring through an un-capped α-ring or differently sized gaps of the CP (Arkinson et al., 2024).

Besides ubiquitin-dependent proteasomal degradation by the 26S proteasome, proteins can also undergo ubiquitin-independent degradation mediated by the 20S proteasome. For example, under certain stress conditions, the 20S proteasome can also degrade proteins independently of ubiquitin and the 19S regulatory particle (Sahu et al., 2021). Approximately 20% of the cellular proteome is estimated to be processed independently of ubiquitination. (Baugh et al., 2009). In many cases, this pathway results in partial rather than complete degradation, preferentially targeting unfolded or intrinsically disordered regions while leaving stable domains intact (Baugh et al., 2009). The growing recognition of ubiquitin-independent proteasomal degradation has substantially expanded the classical view of UPS function. This alternative degradation route appears particularly relevant during proteotoxic stress, when rapid elimination of damaged proteins may be required before ubiquitination can occur.

The 20S proteasome can degrade intrinsically disordered proteins such as αSyn. However, the exact mechanisms by which ubiquitin-independent degradation is regulated is not yet fully understood (Tofaris et al., 2001; Alvarez-Castelao et al., 2014). Targets of this pathway include proteins containing either specific C-terminal degrons (Makaros et al., 2023) or exposed hydrophobic residues (Kisselev et al., 2002) that facilitate proteasomal degradation. This pathway is particularly relevant under conditions of oxidative stress, where damaged or misfolded proteins can directly enter the catalytic chamber without prior ubiquitination or ATP-dependent unfolding (Kisselev et al., 2002).

The 20S proteasome frequently associates with proteasomal activators such as PA200 or PA28, which induce partial gate opening of the α-ring pore complex to regulate substrate access and prevent uncontrolled proteolysis (Sadre-Bazzaz et al., 2010). Importantly, PA200 and its yeast ortholog Blm10 have been linked to the degradation of several neurodegeneration-associated proteins, including mutant huntingtin (Aladdin et al., 2020), tau-441 (Dange et al., 2011) and αSyn (Ali et al., 2026). These findings suggest that ubiquitin-independent proteasomal degradation may represent an important protective pathway in neurodegenerative diseases. Collectively, these findings challenge the traditional view that impaired UPS function necessarily reflects global proteasome failure. Instead, increasing evidence suggests that selective remodeling of proteasome populations, including altered utilization of alternative activators such as PA200/Blm10, may represent an adaptive response to proteotoxic stress. However, it still remains unclear, whether this remodeling is sufficient to counteract progressive αSyn accumulation during human disease. Most supporting evidence currently derives from biochemical studies and experimental model systems, emphasizing the need for validation in human neurons and patient-derived tissues or cell lines. The interplay between neurodegeneration-associated proteins and the ubiquitin-(in)dependent proteasomal degradation will be discussed in greater detail in the following chapter.

## 4 α-Synuclein is both a substrate and an inhibitor of the proteolytic system

### 4.1 Degradation of α-synuclein

The accumulation of misfolded and aggregated αSyn reflects proteostasis dysfunction in synucleinopathies and contributes directly to cellular toxicity in PD. Under physiological conditions cells rely on autophagy and the UPS as primary proteolytic systems. αSyn turnover is maintained by the coordinated action of both pathways, where an impairment of either pathway promotes the accumulation of toxic αSyn species and establishes a self-amplifying cycle of proteotoxic stress. Autophagy is a bulk degradation process that degrades proteins, pathogens, and entire organelles (Parzych and Klionsky, 2014). In contrast, the UPS is a more selective, exclusively proteolytic system that degrades misfolded and short-lived proteins, such as transcription factors (Müller and Hoppe, 2024; Larsen et al., 2026). Notably, both systems are involved in αSyn turnover. The UPS predominantly degrades monomeric, soluble αSyn, whereas autophagy is generally considered to be more efficient in clearing αSyn oligomers and aggregates (Webb et al., 2003; Popova et al., 2015). Importantly, both pathways are impaired in PD and related synucleinopathies (Fellner et al., 2021; Sahoo et al., 2022). Importantly, the relative contribution of UPS and autophagy is not static but changes depending on disease progression and different forms of PD. Under physiological conditions, both systems cooperate to maintain αSyn homeostasis, whereas progressive accumulation of misfolded αSyn increasingly overwhelms their degradative capacity. Consequently, the predominant degradation pathway not only depends on the biochemical properties of αSyn but also on the functional status of the proteolytic network itself. This dynamic interplay represents a central concept for understanding the reciprocal relationship between αSyn and proteostasis.

**FIGURE 3:**
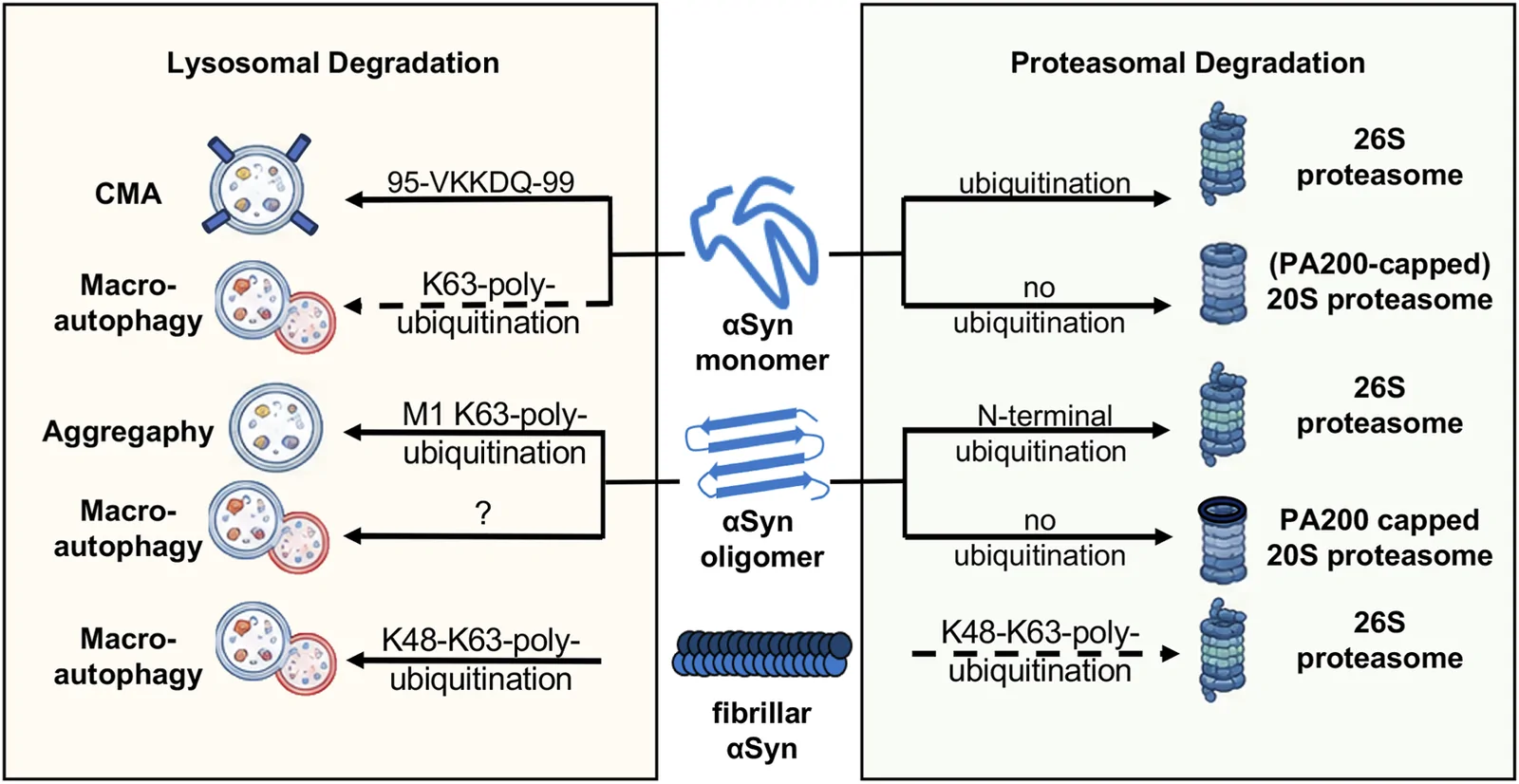
The PD-related protein α-Synuclein as a substrate of cellular proteolytic systems. Degradation pathways of αSyn at different aggregation stages. Dashed arrows indicate pathways that are less favored than the alternative degradation routes. Monomeric αSyn is degraded by the proteasome through both ubiquitin-dependent and -independent mechanisms. The intrinsic 95-VKKDQ-99 motif facilitates degradation by chaperone-mediated autophagy (CMA). The turnover of αSyn oligomers relies predominantly on autophagy; however, both 26S proteasome and PA200-capped proteasome can degrade the αSyn oligomers. Cells retain only a limited capacity to clear fibrillar αSyn; nevertheless, macroautophagy, and to a much lesser extent, proteolysis by the 26S proteasome can mediate turnover of a fraction of fibrillar αSyn.

Under physiological conditions monomeric αSyn is present within the cells, whereas the presence of aggregated αSyn species already indicates a transition into pathology. As such, degradation of monomers represents the predominant physiological route of αSyn turnover. Yet, cells possess a limited capacity to deal with aggregated αSyn species, despite the inhibitory effects they exert on the proteolytic pathways responsible for their degradation (Figure 3). The fate of αSyn is influenced by its various ubiquitination sites and motifs. Proteins smaller than 150 amino acids in length, including αSyn, can be degraded by the proteasome following mono-ubiquitination (Rott et al., 2011; Shabek et al., 2012; Abeywardana et al., 2013). However, this signal does not appear to be universal, as the specific site of ubiquitination significantly impacts degradation efficiency (Abeywardana et al., 2013). Ubiquitinated lysine residues closer to the N-terminus (K6, K12, K23, K32) promote more efficient degradation by the proteasome compared to those closer to the C-terminus (Ho and Wing, 2024). In contrast, chemically synthesized di- and tetra-ubiquitinated αSyn can be efficiently degraded by purified 26S proteasomes, whereas mono-ubiquitin is cleaved off prior to the degradation of the protein, hampering the turnover (Haj-Yahya et al., 2013). The 26S proteasome is incapable of degrading pathological αSyn at its different aggregation states *in vitro* (Zhang et al., 2008; Ali et al., 2026), while others report the opposite (Emmanouilidou et al., 2010; Ye et al., 2020). Such apparently contradictory findings potentially reflect substantial differences in experimental designs, rather than mutually exclusive biological mechanisms. Studies differ considerably with respect to the αSyn species examined (monomers, soluble oligomers, mature fibrils, or ubiquitinated assemblies). Furthermore, the source and composition of the proteasome, protein preparation protocols, ubiquitination status, and *in vitro* assay conditions can vary. Distinct αSyn conformers may exhibit markedly different accessibility to proteasomal engagement and unfolding, something which has been postulated prior, proposing the accessibility of the αSyn C-terminus as a key driver of proteasomal impairment (Suzuki et al., 2020). Consequently, current evidence supports the view that proteasomal degradation of αSyn is highly context-dependent rather than uniformly efficient or inefficient. Canonical ubiquitin-dependent proteasomal degradation requires recognition of the tagged cargo by the 19S RP, followed by substrate unfolding and translocation into the 20S catalytic core. In contrast, 20S can degrade smaller and intrinsically disordered proteins independently of ubiquitination and without requiring the 19S RP (Ben-Nissan and Sharon, 2014). αSyn represents an ideal substrate for ubiquitin-independent degradation as relatively small and highly disordered protein. Accordingly, proteasomal inhibition in SH-SY5Y cells results in increased levels of ubiquitin-free αSyn in the cytoplasm and increased formation of αSyn aggregates. Moreover, *in vitro*, αSyn can be degraded by the 20S proteasome without prior ubiquitination (Tofaris et al., 2001). Recent work supports this observation, showing that both *in vivo* and *in vitro*, the 20S proteasome can turnover αSyn. This degradation is more efficient if the 20S core particle is capped by the proteasome-interacting protein Blm10/PA200. Both assemblies can effectively degrade αSyn without the need for prior ubiquitination (Ali et al., 2026). These findings challenge the traditional view that αSyn degradation depends primarily on canonical ubiquitin-dependent processing by the 26S proteasome. Instead, intrinsically disordered proteins such as αSyn appear to exploit alternative proteasomal degradation routes that bypass ATP-dependent unfolding. This distinction may become particularly important during proteotoxic stress, when ubiquitination capacity or 26S proteasome activity is compromised. Although these observations identify PA200/Blm10-capped proteasomes as promising mediators of αSyn clearance, current evidence remains largely limited to biochemical experiments and model organisms. Whether similar degradation mechanisms substantially contribute to αSyn turnover in human neurons under physiological conditions or during disease progression remains to be established.

Besides proteasomal degradation, monomeric αSyn can also be degraded through different autophagy pathways. The UPS and autophagy work in tandem and are able to compensate each other during proteotoxic stress or impaired proteolysis (Ji and Kwon, 2017; Park et al., 2024). Accordingly, inhibition of the proteasome enhances autophagic αSyn degradation and *vice versa* (Yang et al., 2013). Autophagy is an evolutionarily conserved process that can be categorized into macroautophagy, chaperone-mediated autophagy (CMA), and microautophagy (Parzych and Klionsky, 2014). All three types of autophagy end with the transport of the cargo into the lytic compartment, leading to enzymatic degradation and subsequent release of nutrients into the cytosol. Macroautophagy requires the formation of a phagophore, commonly derived from the ER of the cells and the presence of an LC3-interacting region (LIR), such that substrates can be recognized by the autophagy receptors (Reggiori and Klionsky, 2013; Adriaenssens et al., 2022; Nähse et al., 2024). CMA and microautophagy do not require the formation of a phagophore, as the substrates is delivered directly to the lysosome, which is facilitated by chaperones in the case of CMA (Parzych and Klionsky, 2014). Among these pathways, CMA represents the major route for degradation of soluble αSyn monomers in neurons due to the presence of the KFERQ-like motif 95-VKKDQ-99 within the C-terminal region (Vogiatzi et al., 2008). Macroautophagy is also able to degrade the monomeric αSyn protein, either due to K63 linked ubiquitin chains (Tofaris et al., 2011) or as a result of blocked CMA, which can be caused by dopamine linked αSyn (Martinez-Vicente et al., 2008; Birnbaum and Yue, 2025). This degradation pathway is inhibited by familial mutants of αSyn A53 T and A30 P (Martinez-Vicente et al., 2008). Interestingly, in microglial cells the degradation of αSyn seems to be different, in comparison to neuronal cells. After endocytosis of extracellular soluble αSyn, glial cells subject the protein to selective autophagy. This is achieved by the Toll-like receptor 4 (TLR4)-NF-κB pathway, leading to an activation of the p62 autophagy receptor (Choi et al., 2020).

Oligomeric αSyn is capable of blocking both the UPS and the CMA, which requires other degradation processes to take over the loss of proteolytic capabilities (Lindersson et al., 2004; Cuervo et al., 2004; Martinez-Vicente et al., 2008; Nonaka and Hasegawa, 2009; Popova et al., 2021). The oligomeric species of αSyn represent a highly diverse transition state from monomers to fibrils in size and structure with distinct cytotoxic features (Winner et al., 2011; Emin et al., 2022). The oligomers of αSyn do not represent distinct biochemical entities but rather comprise a heterogeneous population of assemblies that differ substantially in size, stability, surface hydrophobicity, and toxicity. This structural heterogeneity is likely one of the major reasons why different studies report divergent degradation efficiencies and proteasomal interactions. Consequently, conclusions drawn for one oligomer preparation cannot necessarily be generalized to all oligomeric αSyn species. Oligomers need to be removed from cells by autophagy (Lee et al., 2004) or through the 26S proteasome (Emmanouilidou et al., 2010; Ye et al., 2020). The accumulation of αSyn induces aggrephagy as a process to clear protein aggregates through autophagy (Ho and Wing, 2024). Aggrephagy is induced by a specific ubiquitin labelling, facilitated by K63 branched ubiquitin chains attached either to M1 by the LUBAC (Furthmann et al., 2023) or lysine residues by NEDD4 (Tofaris et al., 2011). Either is a prerequisite for the interaction with the autophagy receptor p62. This results in phase separation, the formation of the autophagosome and subsequent fusion with the lysosome (Sun et al., 2018; Kageyama et al., 2021). Specific αSyn oligomers are associated with and degraded by the 26S proteasome (Emmanouilidou et al., 2010). N-terminally ubiquitinated αSyn and the resulting ubiquitinated oligomers can be degraded by the 26S proteasome *in vitro* (Ye et al., 2020). Recent experiments revealed that Blm10/PA200 capped 20S proteasomes are able to degrade smaller oligomeric assemblies (∼70 kDa) of αSyn *in vitro* (Ali et al., 2026).

The treatment of human cells with αSyn pre-formed fibrils (PFF) revealed that the clearance of fully formed αSyn fibrils is problematic for cells. This causes a transient inhibition of autophagy (Gao et al., 2019) as well as UPS (Lindersson et al., 2004; Suzuki et al., 2020). In contrast, inducing autophagy by the addition of rapamycin does prevent formation of αSyn inclusions, whereas autophagy blockage causes the appearance of more inclusions (Gao et al., 2019). The degradation of αSyn fibrils occurs predominantly in glial cells, rather than neuronal cells, similarly to what has been described regarding the autophagic degradation of monomeric αSyn by glial cells (Karim et al., 2026). αSyn fibrils which have been internalized by the SH-SY5Y cells are poly-ubiquitinated by K48-K63 branched chains. This leads to the degradation of ubiquitinated fibrils by both major proteolytic pathways (Gerez et al., 2019).

In recent years a novel, albeit less common, process has been documented. The expulsion of large (∼4 µm) membrane vesicles called exophers was originally described in *C. elegans*. This represents an alternative pathway how cells get rid of toxic aggregates (Melentijevic et al., 2017). Exophers can contain organelles, aggregates and filamentous protein assemblies (Melentijevic et al., 2017; Arnold et al., 2023). Moreover, this pathway was documented in mammalian cardiomyocytes, making this process an interesting future prospect for research in the context of PD (Nicolás-Ávila et al., 2020).

#### 4.1.1 α-Synuclein as a proteolytic inhibitor

**FIGURE 4:**
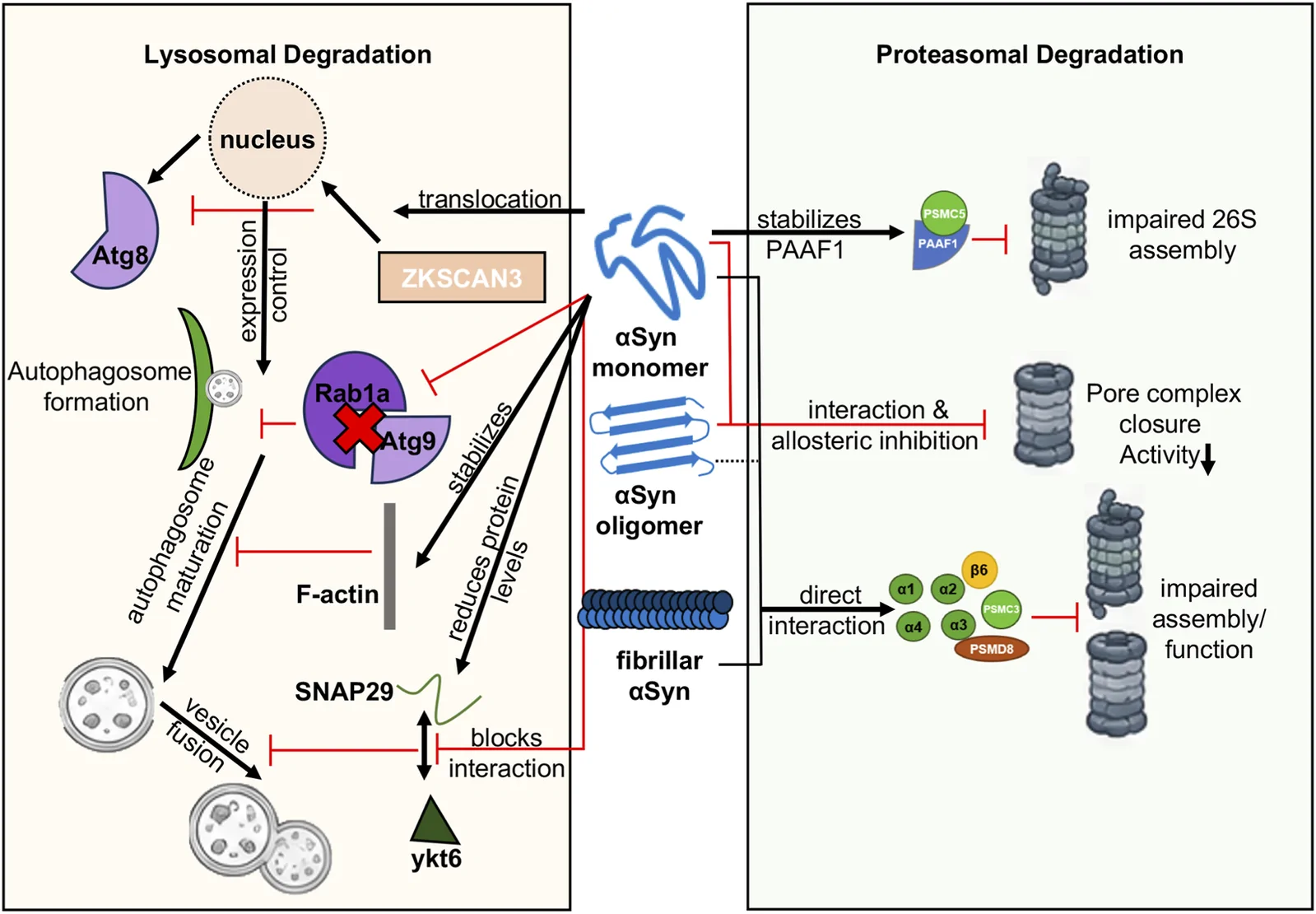
The PD-related protein α-Synuclein as an inhibitor of cellular proteolytic systems. αSyn is an inhibitor of autophagy and proteasomal activity. Outlined are the major mechanism through which αSyn impairs autophagy as well as proteasomal protein degradation. The αSyn mutant A30 P promotes nuclear import of the autophagy repressor ZKSCAN3, reducing the expression levels of ATG8 and overall lowering autophagy. In addition, inhibition of Rab1a results in mis-localization of Atg9, thereby impairing the formation of autophagosomes. Stabilization of the cytoskeleton, particularly F-actin, impairs autophagosome maturation, transport and fusion with the lysosome. The latter aspect is compounded by reduced levels of the SNARE protein SNAP29 and by impaired interaction between SNAP29 and ykt6, further inhibiting the autophagosome-lysosome fusion. Phosphorylated αSyn stabilizes the regulatory particle (RP) base chaperone PAAF1, thereby impairing 26S proteasome assembly and reducing overall proteolytic potential. In addition, the interaction of A11-positive αSyn oligomers, as well as αSyn monomers with the proteasome results in significant reduction of proteasome activity, likely through stabilization of a closed-gate conformation. Lastly, direct interactions between proteasomal subunits and αSyn monomers, fibrils and potentially oligomers have been shown. However, whether these interactions primarily impair proteasome assembly, catalytic activity, or both remains to be elucidated.

The PD-related protein αSyn has been reported to impair both major proteolytic pathways at various stages of aggregation. Inhibition of the proteasome occurs through multiple mechanisms, whereas autophagy is impaired either by inhibition of autophagosome formation, or by interference with autophagosome maturation and its fusion with lysosomes. The following section summarizes the mechanisms underlying autophagy impairment and then focuses on direct effects of αSyn on proteasome function. Analysis of post-mortem brains of PD patients provides evidence for dysfunctional macroautophagy and chaperone-mediated autophagy (Fellner et al., 2021). Impairment of autophagy as a result of aberrant αSyn and its aggregates results in a lack of turnover of monomeric and dimeric αSyn. Over time, this leads to a progressive loss of proteolytic capacity within neurons. This creates a self-propelling cycle of αSyn accumulation (Martinez-Vicente and Vila, 2013; Tanik et al., 2013). Age-associated decline in autophagic efficiency may further sensitize neurons to αSyn-mediated proteotoxicity. Initiation of macroautophagy is impaired by overexpression of the αSyn-coding gene *SNCA* in both human cell models and mice (Winslow et al., 2010). *SNCA* overexpression inhibits Rab1a, which results in an Atg9 mis-localization and consequently impaired autophagosome formation (Figure 4). A more widespread inhibition of autophagy has been observed for the A30 P αSyn mutant. Expression of the A30 P variant in midbrain dopaminergic neurons resulted in an increased trafficking of the master transcriptional repressor ZKSCAN3 into the nucleus (Chauhan et al., 2013; Lei et al., 2019) (Figure 4). This re-localization leads to reduction of LC3 levels and subsequent reduction in autophagy. In addition, extracellular αSyn uptake reduces LC3 levels in microglia through activation of TLR4 and subsequent reduction of LC3-II levels (Tu et al., 2021).

The interference of pathological αSyn with autophagosome trafficking and autolysosome fusion has been extensively investigated (Figure 4). Reduced capability of vesicle fusion can be in part attributed to reduced levels of the SNARE protein SNAP29 in the presence of increased αSyn levels (Tang et al., 2021). This leads to an altered SNARE-complex composition and interferes with autolysosome fusion. This defect is further exacerbated by reduced association between ykt6 and SNAP29 in neurons derived from induced pluripotent stem cells (iPSC) (Pitcairn et al., 2023). The protein ykt6 plays a pivotal role in autolysosome fusion (Matsui et al., 2018). Before autophagosome and lysosome fuse, they must be transported into close spatial proximity, a process facilitated by the cytoskeleton. In particular, F-actin plays a significant role in selective autophagy (Lee J. Y. et al., 2010) and is negatively influenced in αSyn pathology. Work in *Drosophila*, mice and human cells demonstrated that αSyn stabilizes the actin cytoskeleton, especially F-actin. As a result, autophagosome maturation, transport, and fusion with the lysosome are impaired (Sarkar et al., 2021). Pathological αSyn further interferes with vesicle trafficking of the lysosome, reducing the overall hydrolase activity in iPSC derived midbrain neurons (Mazzulli et al., 2016). In mammalian cells, pathological αSyn alters lysosome morphology and causes an increased localization at the plasma membrane. This mis-localization reduces lysosomal functionality and facilitates transfer of lysosomes into neighboring healthy cells, where the αSyn cargo may act as seeding structures and facilitates pathology spreading (Senol et al., 2021). Collectively, these findings demonstrate that αSyn impairs autophagy at multiple stages, including autophagosome formation, vesicle trafficking, autolysosome fusion, and lysosomal function. However, the extent to which autophagy impairment represents an initiating event or a secondary consequence of αSyn accumulation remains yet unresolved. Whereas experimental models clearly demonstrate that increased αSyn levels can disrupt multiple autophagic steps, human pathological studies mainly provide evidence of association rather than direct causality. This distinction is particularly important because aging itself reduces autophagic capacity and may create a cellular environment in which otherwise manageable αSyn levels become increasingly toxic.

The loss of autophagy is compounded by a simultaneous inhibition of the proteasome, drastically reducing the cells proteolytic capabilities. Numerous studies support a significant loss of proteasome activity in the presence of different αSyn species (Snyder et al., 2003; Lindersson et al., 2004; Zhang et al., 2008; Emmanouilidou et al., 2010; Ali et al., 2026) (Figure 4). Snyder et al. (2003) originally showed that aggregated αSyn is a potent inhibitor of 26S activity, whereas it is far less effective as an inhibitor of the 20S activity. *In vitro*, aggregated αSyn inhibited both ubiquitin-dependent and independent 26S activity at sub-nanomolar concentrations. In contrast, αSyn monomers failed to inhibit 26S ubiquitin-dependent 26S activity and only had a minor effect on 26S ubiquitin-independent activity at millimolar concentrations. Whereas αSyn aggregates are efficient inhibitors of the 26S proteasome, their inhibitory efficacy is significantly reduced when analyzing 20S activity. These observations provided the initial framework for the concept that aggregated αSyn directly suppresses proteasomal function. However, subsequent studies have demonstrated that the inhibitory capacity of αSyn strongly depends on the biochemical properties of the aggregated species examined. Therefore, rather than representing a general feature of all αSyn aggregates, proteasome inhibition appears to be associated with specific conformational states or oligomeric assemblies. As outlined before, the heterogeneity of αSyn and its succeeding aggregates can result in significantly differing experimental outcomes. Moreover, the impact of monomeric αSyn on 20S activity remains unclear, as different studies showed either a strong inhibitory potential (Snyder et al., 2003), or a significantly reduced effect (Lindersson et al., 2004). Such directly opposing results might be due to different purification methods when it comes to αSyn and the proteasome, as well as the used protein concentrations and buffers for *in vitro* analysis, rather than being solely rooted in biology, as monomeric αSyn does not show the same polymorphisms we can observe in oligomers and fibrils.

Some of the observations were brought into question by later reports, indicating that neither αSyn protofibrils, nor monomers or dimers are capable of inhibiting the peptidase activity of the 26S proteasome *in vitro* (Zhang et al., 2008). Instead, αSyn protofibrils inhibited the degradation of unfolded (ubiquitin-independent) and folded (ubiquitin-dependent) proteins, when present at high molar excess relative to the 26S proteasome. Similar effects were observed for monomeric and dimeric αSyn, although only at concentrations unlikely to be physiologically relevant. The authors proposed that binding of αSyn protofibrils to the 26S proteasome as well as to its substrates might be the reason for reduction of protein degradation. In addition, αSyn oligomers have their own distinct inhibitory effects on 26S proteasomes in cell extracts from human cell cultures and mice brains, although the precise molecular mechanisms remain still elusive (Emmanouilidou et al., 2010). Further *in vitro* studies into 20S activity under αSyn proteotoxic stress partially contradict the work by Snyder et al. (Lindersson et al., 2004). The authors show that monomeric αSyn is a substantially weaker inhibitor of 20S activity, when compared to fibril species. Moreover, they show that soluble αSyn oligomers are also efficient inhibitors of 20S activity, likely due to a conserved structural motif shared with fibrils. Importantly, Lindersson et al. reported selective inhibition of chymotrypsin-like activity, whereas trypsin- and caspase-like activities of the proteasome remained largely unaffected. More recently, it has been shown that oligomeric αSyn, Aβ and Huntingtin are strong inhibitors of 20S proteasomal activity, specifically the chymotrypsin-like activity (Thibaudeau et al., 2018) (Figure 4). The similar effects of the mentioned proteins, indicates that the structural motif present within oligomers and fibrils is not unique to αSyn, but rather a shared features amongst the tree. Thus, the presence and accessibility of such a feature as a result of different preparational and experimental conditions, would significantly influence the results, potentially contributing to the observed differences.

As oligomers encompass a variety of different, in our case αSyn, assemblies, recent work has been focused on A11-positive oligomers, as key drivers of proteasomal impairment (Thibaudeau et al., 2018; Ali et al., 2026). A11 antibody recognizes oligomeric assemblies of several amyloidogenic proteins associated with neurodegenerative diseases (Glabe, 2004). The ability to recognize different amyloid proteins in their oligomeric form reinforces the idea of a shared motif between the amyloid proteins even at early aggregation states, which likely persists throughout amyloidogenesis. The identification of shared structural features among amyloidogenic oligomers suggests that proteasome inhibition may represent a broader mechanism of proteotoxic stress rather than an αSyn-specific phenomenon. Nevertheless, whether A11-positive structures represent the predominant toxic species in human PD remains unclear, as oligomer populations detected experimentally may not fully reflect the diversity of assemblies present in patient tissue. This putative shared structural motif appears to be directly involved in the inhibition of 20S and 26S chymotrypsin activity. Support for this hypothesis comes from the observation that Aβ-iO oligomers fail to inhibit the proteasome activity when co-incubated with A11 antibodies (Thibaudeau et al., 2018). A comparable phenomenon has been previously described for αSyn oligomers. Treating cell lysates with the amyloid interacting dye Congo Red prior to purifying different αSyn species disrupted the formation of proteasome-interacting αSyn oligomers, thereby rescuing the proteolytic activity of the complex (Emmanouilidou et al., 2010). Some of the contradictory findings regarding αSyn-mediated inhibition on different proteasome assemblies could lie in the inherent polymorphism of αSyn fibrils (Mehra et al., 2021). This conformational diversity provides a plausible explanation for apparently conflicting observations across experimental systems. αSyn fibrils generated *in vitro* under different conditions can vary substantially in molecular architecture, exposed surfaces, and interaction partners, potentially leading to different effects on proteasome function. Consequently, the inhibitory activity measured in biochemical assays should not automatically be extrapolated to disease-associated fibrils present in human neurons. *In vitro,* different cations and varying buffer compositions generate structurally distinct αSyn fibrils, that differ from the fibril structure found in the brains of PD patients (Yang et al., 2022). A 2020 study revealed that the C-terminal region of αSyn plays a major role in proteasomal inhibition (Suzuki et al., 2020). The authors proposed that different αSyn fibrils, where the C-terminus remains unfolded as a “fuzzy coat”, differentially inhibit the proteasomal activity, depending on the accessibility of the C-terminus for proteasome interaction. Specifically, they outline that the presence KCl “masks” the C-terminal region of αSyn, reducing the interaction with the proteasome and diminishing the inhibitory effect. How this would translate to αSyn monomers or oligomers remains to be investigated. However, these findings suggest that the C-terminal region of αSyn is an important determinant of proteasome interaction and inhibition.

A potential link between direct interaction of αSyn and proteasomal subunits has long been investigated as a mechanism underlying proteasomal inhibition (Figure 4). Distinct αSyn interaction partners within the proteasome complex could be identified. αSyn interacts to the α-ring protein PSMA3 of the core particle (CP) (Alvarez-Castelao et al., 2014). The N-terminus of αSyn interacts to the negative C-terminus of PSMA3, which contributes to the 20S ubiquitin-independent degradation of αSyn monomers. This acts as a “gate-opener” and might also include contributions from other α-ring subunits. Filamentous αSyn interacts with multiple proteasomal subunits, including the α-ring subunits PSMA1, PSMA2, PSMA3, and PSMA4, as well as the catalytic subunit PSMB6, potentially inducing the loss of proteasomal activity. (Lindersson et al., 2004). Less abundant interactions were detected with the regulatory particle (RP) subunits PSMC3 and PSMD8. Moreover, studies from multiple groups have shown that both αSyn fibrils and monomers interact with RP subunits, such as PSMC4, which has been directly linked to reduced 26S proteasome activity (Snyder et al., 2003). Screening approaches using rat brain tissue further revealed interactions between αSyn and PSMC3 of the RP base, although the functional consequences for proteasome activity were not assessed (Ghee et al., 2000).

Recent work has additionally identified an interaction between αSyn and the Rpt2 base subunit, the yeast homolog of the PSMC1 ATPase, suggesting that αSyn may interfere with substrate unfolding, translocation, or 26S proteasome assembly (Popova et al., 2021). This is particularly noteworthy given the close physical proximity of the AAA + -ATPases PSMC1, PSMC4, and PSMC3 within the RP base (Lander et al., 2012). These ATPases are essential for substrate unfolding and translocation into the catalytic chamber of the 20S proteasome. Consequently, interactions between αSyn and these subunits could impair their function, potentially resulting in a “clogged” and inactive 26S proteasome. In addition, the base subunit PSMC3 binds to the α-ring of the core particle (CP) between PSMA1 and PSMA3 (Tian et al., 2011), with PSMA3 also identified as an interaction partner of αSyn. Interactions within this pore-forming region may therefore contribute to stabilization of the closed-gate conformation of the 20S proteasome observed in the presence of αSyn oligomers (Thibaudeau et al., 2018). Collectively, these findings indicate that αSyn interacts with both catalytic and regulatory proteasome subunits, potentially impairing substrate recognition, ATPase-driven unfolding, gate dynamics, and proteasome assembly. However, direct interactions alone do not necessarily establish functional inhibition. Many reported αSyn-proteasome interactions have been identified using biochemical approaches, overexpression systems, or affinity-based assays, and the physiological relevance of individual interactions remains to be validated. Future studies combining structural approaches with endogenous expression systems will be important to determine which interactions occur during disease progression.

Furthermore, αSyn can directly interfere with the assembly of the 26S proteasome (Figure 4). αSyn physically interacts and stabilizes as well chaperone Rpn14 which is required for 26S assembly in yeast, as is the human counterpart PAAF1 in human cells. This induces reduced cellular 26S proteasomal activities in both cell types (Galka et al., 2024). Rpn14 binds the yeast ATPase Rpt6 (PSMC5 in humans), and escorts it to the CP during assembly of the RP base. Stabilization of Rpn14 by αSyn increases intracellular Rpn14 abundance and likely impairs correct base assembly, thereby reducing 26S proteasome formation.

Another mechanism through which αSyn may impair proteasomal activity involves alterations in proteasomal subunit abundance or proteasome composition. Earlier studies suggested that expression of wild-type or mutant αSyn leads to changes in the abundance of proteasome associated proteins, whereas the overall abundance of proteasomes within the cell remains unchanged (Chen et al., 2005). Recent reports identified pS129-dependent alterations in proteasomal subunit levels (Popova et al., 2021). Interestingly, CP subunits PSMA1, PSMA2, PSMA3 and to a much lesser extent RP subunits PSMD8, have been identified as directly interacting with the αSyn fibrils, possibly contributing to the aberrant abundance within cells (Lindersson et al., 2004). These findings suggest that pathological αSyn may impair proteasome function not only through direct inhibition, but also by altering proteasome assembly dynamics and subunit homeostasis. These mechanisms introduce an important distinction between acute proteasome inhibition and long-term proteostasis remodeling. αSyn may rather gradually alter proteasome composition by interfering with the assembly pathways than directly blocking catalytic activities. Such remodeling could have broader consequences for cellular protein quality control, particularly in long-lived neurons with limited regenerative capacity.

In summary, αSyn impairs proteolytic systems through multiple converging mechanisms, including direct proteasome inhibition, disruption of autophagy, altered vesicle trafficking, interference with proteasome assembly and dysregulation of proteasomal subunit abundance. Importantly, these effects are highly dependent on αSyn conformation, aggregation state, and cellular context. Current evidence supports a model in which pathological αSyn and impaired proteostasis reinforce each other in a self-amplifying cycle; however, the relative contribution of individual mechanisms to human disease progression remains unresolved. Most mechanistic insights originate from biochemical studies, cellular models, and animal systems, emphasizing the need for further validation in patient-derived neurons and human pathological material. Aging-associated decline in proteostasis likely further sensitizes neurons to these effects, thereby promoting the transition from physiological αSyn turnover to persistent proteotoxicity and neurodegeneration.

## 5 Harnessing proteolysis for novel therapeutic approaches

Restoring proteolysis impaired by αSyn pathology as a therapeutic approach has been investigated in the context of UPS, proteasome activity as well as autophagy. Such approaches are conceptually attractive, yet practical solutions remain limited. On the bright side, many proteotoxic effects can be ameliorated by comparatively modest reduction of disease causing protein levels (Dantuma and Bott, 2014), which, for instance, could be achieved by reducing gene expression, or ameliorating protein levels. In this section, we focus on strategies that restore autophagy and UPS function, thereby enhancing αSyn clearance and proteostasis.

### 5.1 Autophagy-based approaches

Clearance of aggregated or misfolded αSyn proteins through autophagy has garnered increasing interest over the past years. Many current efforts focus on restoring normal autophagic function within cells, effectively countering the inhibition resulting from αSyn-cytotoxicity, to effectively hydrolyze αSyn aggregates.

Induction of autophagy through inhibition of the mammalian target of rapamycin (mTOR), especially mTORC1, is one of the best-established approaches. mTORC1 is a central regulator of cellular growth and metabolism that promotes anabolic processes while inhibiting autophagy. Its sustained activation is linked to aging and age-related decline in proteostasis, while inhibition promotes autophagy. Treatment of cell cultures and 1-Methyl-4-phenyl-1,2,3,6-tetrahydropyridine (MPTP) mice PD models with rapamycin have revealed clear neuroprotective effect (Malagelada et al., 2010). This rescue effect coincides with partial mTOR inhibition, whereas complete catalytic inhibition does not yield the same outcome. However, changes in αSyn degradation were not investigated. A subsequent study using MPTP mice and the rapamycin analogue temsirolimus demonstrated that autophagy is upregulated as a result of inhibited mTOR following the treatment (Siracusa et al., 2018). This increase in autophagy resulted in reduced αSyn levels and partially ameliorated the behavioral effects associated with MPTP-induced parkinsonism. Rapamycin and its analogues have a major drawback, as they affect numerous cellular processes beyond autophagy, including apoptosis and cytoskeleton dynamics. In addition, long term treatment also carries serious side-effects such as immunosuppression (Zhang et al., 2025). Thus, despite promising results in experimental models, mTOR inhibition illustrates the central challenge of targeting autophagy therapeutically that limits long-term application. Furthermore, most studies demonstrating reduced αSyn burden have been performed in toxin-based or transgenic animal models, and whether these findings translate into clinically meaningful disease modification in human PD remains yet unknown.

The use of natural compounds to activate autophagy has been studied as well. Caffeine protects against αSyn-induced cytotoxicity by restoring autophagic activity (Luan et al., 2018). Similarly, corynoxine, an alkaloid isolated from plants, induces autophagy in mice with rotenone-induced parkinsonism, also leading to reduced neuroinflammation (Chen et al., 2021). Corynoxine activates autophagy in an mTOR dependent manner. Another compound isolated from plants, piperine, has been shown to reduce motor symptoms in transgenic mice, due to an increased autophagic degradation of αSyn (Liu et al., 2016; Li et al., 2022). Notably, these compounds act through distinct mechanisms, highlighting the diversity of pharmacological approaches available for autophagy modulation. However, many naturally derived compounds face substantial translational limitations, including unclear molecular targets, variable bioavailability, rapid metabolism, and uncertain penetration into the central nervous system. Thus, although these compounds provide valuable mechanistic insights, their therapeutic potential requires careful future validations in models that better reflect human disease.

AMP-activated protein kinase (AMPK) represents another important regulator of autophagy and acts as an antagonist to mTOR. AMPK functions indirectly by inhibiting mTOR suppressors through phosphorylation. AMPK is inactivated in proliferating neuronal cells, contributing to the autophagosomal impairment (Dulovic et al., 2014). Activation of AMPK by pharmacological agents has neuroprotective effects, some of which appear independent of autophagy. Prior observations with the compound resveratrol, which can be isolated from red grapes, enhances the degradation of αSyn by autophagy induction in an AMPK-dependent manner (Wu et al., 2011).

Another important player involved in autophagy that has been targeted by pharmacological compounds is Beclin-1, which is involved in the nucleation of autophagosomes. The small molecule KYP-2407 increases the degradation of aggregated αSyn (Rostami et al., 2020). This increase supports the clearance of A30 P αSyn in mice, mediated by adjusting the expression of Beclin-1 (Savolainen et al., 2014). Beclin-1 dependent, but mTOR independent, induction of autophagy by isorhynchophylline resulted in the autophagic degradation of αSyn (Lu et al., 2012). Overexpression of the lysosomal-associated membrane protein 2a (LAMP2a) reduces αSyn levels and partially rescues neurodegeneration (Xilouri et al., 2013). However, the translational feasibility of such gene-based approaches remains uncertain.

mTOR independent activation of autophagy has revealed that lithium protects SH-SY5Y from rotenone induced cytotoxicity (Hou et al., 2015). This protection is dependent on functioning autophagy but does not reveal direct changes in αSyn degradation, rather highlighting a possible alternative way of protecting cells. Trehalose represents another compound that induces autophagy in a mTOR independent manner (Sarkar et al., 2007; Cunha et al., 2022). This increased induction of autophagy leads to an enhanced clearance of αSyn and huntingtin aggregates within mammalian cell models (Sarkar et al., 2007). A more general activation of autophagy has been achieved by the activation of the transcription factor EB (TFEB), either by 2-hydroxypropyl-β-cyclodextrin or overexpression (Song et al., 2014; Arotcarena et al., 2019). TFEB overexpression clearly resulted in increased autophagic clearance of αSyn and reduced aggregation.

Besides altering the levels of autophagy, other compounds have been tested. Rifampicin promotes lysosomal acidification and rescues autophagic flux following rotenone treatment (Liang et al., 2020). Similarly, nanoparticles capable of restoring lysosomal acidification improve lysosomal function and reduce neurodegeneration in MPTP-treated mice (Bourdenx et al., 2016).

Collectively, current evidence suggests that enhancing autophagic flux, restoring lysosomal function, or targeting key autophagy regulators can reduce αSyn burden and ameliorate neurodegenerative phenotypes in experimental models. However, most approaches remain preclinical, and their long-term efficacy and safety in humans remain unclear. Major challenges include achieving sufficient brain penetration, maintaining long-term safety, avoiding excessive activation of degradation pathways, and determining whether intervention at symptomatic disease stages can reverse established αSyn pathology.

### 5.2 UPS-based approaches

The mechanisms through which αSyn impairs proteasome function also reveal potential therapeutic opportunities to restore proteolytic activity. Such approaches are particularly attractive because they may facilitate the selective removal of misfolded or aggregation-prone αSyn species. Compared with global stimulation of proteolysis, strategies aimed at selectively enhancing degradation of pathological αSyn species may offer improved therapeutic potential. However, achieving sufficient substrate specificity remains a major challenge, particularly because αSyn exists in multiple conformational states and several physiological functions of soluble αSyn remain incompletely understood. One strategy is to prevent αSyn aggregation itself, as aggregated species are among the most potent proteasomal inhibitors. Considerable effort is therefore directed toward development of aggregation inhibitors (Peña-Díaz et al., 2023). Targeting existing cellular pathways could be another promising path. Chemically induced overexpression of chaperones has resulted in reduced disease severity. Arimoclomol induces an increased expression of heat shock proteins (Hsp), particularly Hsp70. This leads to a partial rescue of disease symptoms and significantly decreased progression rate in mice models of spinal and bulbar muscular atrophy (SBMA) as well as amyotrophic lateral sclerosis (ALS) (Kalmar et al., 2012; Malik et al., 2013). Similarly, the small molecule YM-1 supports the ability of HSP70 to interact with unfolded substrates and increases the degradation of poly-Q-expanded androgen receptor (AR) in SBMA *Drosophila* flies (Wang et al., 2012). Although pharmacological enhancement of chaperone activity has shown beneficial effects in several protein-misfolding disorders, these findings cannot be directly extrapolated to PD. Differences in disease mechanisms, target proteins, and neuronal vulnerability may strongly influence therapeutic outcomes.

Another approach through which increased proteasomal turnover of proteotoxic proteins can be achieved is by increasing the pool of ubiquitin. αSyn exerts a significantly reduced cytotoxic effect in *Drosophila* neurons when ubiquitin is overexpressed (Lee et al., 2009). The rescue effect is directly linked to K48-linked polyubiquitin associated with proteasomal degradation.

Increasing the overall UPS capacity represents a further strategy. The transcription factors Nrf1 and Nrf2 regulate the capacity of the UPS (Dantuma and Bott, 2014). The naturally occurring compound sulforaphane increases the degradation of mutant huntingtin in mice models and appears to enhance proteasomal activity, despite simultaneously affecting autophagy (Liu et al., 2014). Improving the degradability of proteins linked to neurodegenerative diseases has also been investigated as a potential therapeutic approach. Proteasome-associated deubiquitylating enzymes like USP14 can act as a molecular timer, regulating the window for proteasomal degradation and rescuing weakly ubiquitinated proteins (Lam et al., 1997). Inhibition of USP14 with the small molecule IU1 accelerates proteasomal degradation of aggregation-prone proteins, including those implicated in neurodegenerative diseases (Lee B. H. et al., 2010). Whether this translates into therapeutic benefit *in vivo* remains to be determined.

Direct stimulation of the proteasome activity to increase the turnover of disease-associated proteins has recently attracted considerable attention. Based on the conserved HbYX-motif of proteins interacting with the α-ring of the 20S proteasome, mimetic peptides containing this motif were shown to rescue proteasome activity in the presence of pre-amyloid oligomers (Chuah et al., 2023). A recent study analyzed the effect of Blm10/PA200, 20S interacting proteins, which contain the HbYX-motif and have been shown to modulate 20S activity (Ali et al., 2026). Blm10/PA200 rescued proteasome activity in the presence of αSyn monomers and oligomers, while simultaneously promoting more efficient degradation of monomeric αSyn than uncapped 20S proteasomes and enabling the degradation of αSyn oligomers *in vitro*. Increased αSyn turnover was also observed in yeast and mammalian cell models. These findings identify interference with proteasome remodeling as a promising potentially distinct therapeutic concept compared with conventional proteasome activation. Rather than globally increasing proteolytic activity, selective recruitment of alternative proteasome regulators such as PA200/Blm10 may enhance degradation of specific proteotoxic substrates while preserving basal proteasome functions. However, current evidence remains yet limited to experimental systems, and the feasibility of pharmacologically modulating proteasome composition in the human brain remains to be established.

A more direct method of decreasing the abundance of cytotoxic proteins is their targeted degradation (Dantuma and Bott, 2014). The concept is not limited to proteasomal targeting, as autophagy can also serve as a route for targeted degradation. Hsp90 inhibitors such as 17-AAG and 17-DMAG increase degradation of disease-associated proteins, including polyQ-expanded (AR) (Waza et al., 2005; Tokui et al., 2009). Another way to increase proteasomal turnover of disease related-proteins has been shown using polyQ-expanded AR as well. Increased phosphorylation induced by insulin-like growth factor-1resulted in increased proteasomal turnover of the protein and reduced disease symptoms (Palazzolo et al., 2007).

Possible ways to redirect the UPS to target aggregation-prone proteins have been explored. Engineered chimeric ubiquitin ligases can redirect ubiquitination to specific proteins of interest (Zhou et al., 2000). These ligases typically combine a recognition domain for the target protein with a ubiquitination domain (e.g., RING, HECT, U-box) that catalyzes ubiquitin addition or recruits the substrate to an E3 ligase complex (Zhang and Zhou, 2005). A Dorfin-CHIP chimera can efficiently ubiquitinate mutant protein SOD1 associated with ALS (Ishigaki et al., 2007). The potential for very high specificity of these chimeras is their biggest strong suit but potential off-target effects and regulation issues might exist. Proteolysis-targeting chimeric molecules (PROTACs) represent a related strategy for the future (Kuemper et al., 2024). PROTACs have been applied to αSyn aggregates, directing them towards autophagic degradation due to inability of proteasome to turnover larger aggregates (Lee et al., 2023). Several compounds successfully induce αSyn degradation, although the precise proteolytic pathway involved often remains unresolved (Wen et al., 2023). An important limitation of targeted degradation approaches is the requirement for efficient delivery across the blood-brain barrier and into neurons. Furthermore, targeting aggregated αSyn raises additional challenges because large fibrillar assemblies may not be accessible to the proteasome and may require autophagic clearance. Consequently, future development will require careful optimization of target recognition, degradation pathway selection, pharmacokinetics, and long-term safety.

Overall, restoration of proteostasis represents a promising therapeutic strategy for synucleinopathies, but successful clinical translation will require approaches that go beyond generalized activation of degradation pathways. Rather than targeting αSyn aggregation alone, future interventions will likely need to combine enhancement of autophagy, restoration of proteasome function, and selective degradation of pathological αSyn species. Such multimodal approaches may be particularly important in the aging nervous system, where progressive decline of proteolytic capacity increases susceptibility to αSyn-mediated proteotoxicity. However, nearly all currently discussed approaches remain at the preclinical stage, and substantial challenges related to delivery, specificity, toxicity, and disease timing must be overcome before therapeutic application becomes feasible.

## 6 Concluding remarks

Extensive efforts to elucidate the molecular basis of proteolytic dysfunction in PD and related neurodegenerative disorders have been performed in the last decade. However, significant gaps in our understanding still remain. The remarkable heterogeneity of αSyn species, including distinct conformers, posttranslationally modified variants, and disease-associated strains, complicates the interpretation of experimental findings and most likely contributes to the apparent discrepancies reported across studies. Although clinically grouped under a single diagnosis, PD encompasses a spectrum of phenotypes that may arise from distinct and only partially overlapping molecular mechanisms. Rather than representing conflicting observations, these differences may reflect a biologically meaningful heterogeneity, which underlies the clinical and pathological diversity of synucleinopathies. Recognizing and systematically incorporating this variability into experimental design and data interpretation will be essential for progressing research in the field.

Collectively, the available evidence highlights the intricate and dynamic relationship between αSyn and the cellular proteostasis network. The degradative fate of αSyn is strongly influenced by its conformational state and posttranslational modifications, while pathological αSyn species can progressively compromise both proteasomal and autophagic pathways. This bidirectional interplay places αSyn at the center of a complex proteostasis network whose dysfunction becomes increasingly pronounced during aging and disease progression. Future therapeutic approaches will likely require restoration of global proteostasis rather than targeting αSyn aggregation alone. Strategies combining enhancement of autophagy, restoration of proteasome activity, and selective degradation of pathological αSyn species may prove particularly effective. Numerous fundamental questions remain yet unresolved. It is still unclear which αSyn species represent the most relevant therapeutic targets in human disease, whether proteostasis impairment is primarily a driver or consequence of αSyn pathology, and at which disease stage restoration of degradation capacity would provide meaningful benefit. Addressing these questions will require integration of structural biology, patient-derived cellular models, longitudinal studies, and improved biomarkers that reflect proteostasis dysfunction in living patients. A deeper understanding of how aging alters the interaction between αSyn and cellular proteolytic systems will be essential for the development of disease-modifying therapies for Parkinson’s disease and related synucleinopathies.
